# Sodium Super Ionic Conductor-Type Hybrid Electrolytes for High Performance Lithium Metal Batteries

**DOI:** 10.3390/membranes13020201

**Published:** 2023-02-06

**Authors:** Po-Yu Sung, Mi Lu, Chien-Te Hsieh, Yasser Ashraf Gandomi, Siyong Gu, Wei-Ren Liu

**Affiliations:** 1 Department of Chemical Engineering and Materials Science, Yuan Ze University, Taoyuan 32003, Taiwan; 2 Key Laboratory of Functional Materials and Applications of Fujian Province, Xiamen University of Technology, Xiamen 361024, China; 3 Department of Mechanical, Aerospace, and Biomedical Engineering, University of Tennessee, Knoxville, TN 37996, USA; 4 Department of Chemical Engineering, Massachusetts Institute of Technology, Cambridge, MA 02142, USA; 5 Department of Chemical Engineering, Chung Yuan Christian University, Taoyuan 32023, Taiwan

**Keywords:** composite solid electrolytes, lithium metal batteries, NASICON-type powders, LATP powders, optimal setting

## Abstract

Composite solid electrolytes (CSEs), composed of sodium superionic conductor (NASICON)-type Li_1+x_Al_x_Ti_2-x_(PO_4_)_3_ (LATP), poly (vinylidene fluoride-hexafluoro propylene) (PVDF-HFP), and lithium bis (trifluoromethanesulfonyl)imide (LiTFSI) salt, are designed and fabricated for lithium-metal batteries. The effects of the key design parameters (i.e., LiTFSI/LATP ratio, CSE thickness, and carbon content) on the specific capacity, coulombic efficiency, and cyclic stability were systematically investigated. The optimal CSE configuration, superior specific capacity (~160 mAh g^−1^), low electrode polarization (~0.12 V), and remarkable cyclic stability (a capacity retention of 86.8%) were achieved during extended cycling (>200 cycles). In addition, with the optimal CSE structure, a high ionic conductivity (~2.83 × 10^−4^ S cm^−1^) was demonstrated at an ambient temperature. The CSE configuration demonstrated in this work can be employed for designing highly durable CSEs with enhanced ionic conductivity and significantly reduced interfacial electrolyte/electrode resistance.

## 1. Introduction

Reliable energy storage technologies are needed for future-generation electronic devices (e.g., laptop computers and cellphones), electric vehicles (EVs), and electric grids. Depending on the application, the required attributes for energy storage may vary; however, high power density and capacity utilization, along with enhanced stability, safety, and environmental friendliness, are urgently demanded [1,2]. All solid-state energy-storage devices (e.g., batteries and supercapacitors) with compact designs and light weights are very promising for enabling high energy densities, as well as enhanced safety and thermal stability, since the safety hazards and physicochemical limitations associated with the liquid electrolytes can be mitigated via utilizing solid electrolytes [3,4,5,6,7]. An ideal solid electrolyte is expected to possess a high level of ionic conductivity, a wide electrochemical stability window, and compatibility (chemically and electrochemically) with the electrode materials [4]. 

Several prior efforts have been dedicated to developing various types of Li superionic conductors, including studies that used garnet [8,9], sodium super ionic conductors (NASICON) [10,11,12,13], thio-phosphates [14], argyrodites [15,16,17], and many other types of materials. Among them, lithium-aluminum-titanium-phosphate (Li_(1+x)_Al_x_Ti_(2−x)_(PO_4_)_3_, LATP) with a NASICON-type structure is an encouraging solid-state electrolyte that possesses a relatively high ionic conductivity capability (10^−4^–10^−3^ S cm^−1^ at ambient temperature), along with superior chemical stability [18,19,20,21]. It is well established that LATP ceramics are capable of crystallizing in a rhombohedral lattice consisting of a PO_4_ tetrahedra adjacent to a TiO_6_ octahedra, forming a three-dimensional pathway [22] and enabling lithium-ion conductivity. However, since LATP ceramic particles are brittle, the solid electrolyte/electrode interfacial structure may become unstable due to volumetric changes in the electrode material during lithium-ion intercalation/deintercalation [4]. One strategy for alleviating this potential issue is to design composite solid electrolytes (CSEs) that incorporate ceramic particles within a polymer matrix in order to provide robust mechanical properties [13,23,24]. 

In this work, we designed and fabricated CSEs with LATP crystallites and a polymeric matrix for solid-state Li-metal batteries. It is generally recognized that polymer electrolytes with matrices such as poly(ethylene oxide), poly(vinylidene fluoride) (PVDF), and poly(vinylidene fluoride-co-hexafluoropropylene) (PVDF-HFP), despite possessing great flexibility and superior compatibility (with various electrode configurations), usually suffer from poor ionic conductivity [25,26,27]. Notably, PVDF with strong electro-withdrawing functional groups (–C–F) displays a high dielectric constant (*ε* = 8.4), facilitating the dissolution of Li salts while maintaining high concentrations of charge carriers [28]. Since the copolymerization of two monomers, PVDF-HFP, results in reduced crystallinity compared to the use of pristine PVDF [29], the as-prepared electrolyte structure becomes amorphous and capable of withholding significant amounts of electrolytes. Hence, a PVDF-HFP matrix, as a gel polymer electrolyte, was selected along with the LATP ceramic particles as the CSE.

To further improve the battery performance, an optimal CSE configuration (i.e., Li salt/LATP ratio, thickness, and C content) was engineered for the high-performance Li-metal batteries. The cyclic stability of the as-prepared solid-state Li-metal batteries was also systematically explored, with the CSEs containing a LiFePO_4_ cathode active material. The excellent compatibility of the CSEs with the Li anodes and its effectiveness in suppressing the Li dendrite growth confirmed that it had a robust battery structure, with low inner resistance and superior capacity retention during long-duration cycling. Accordingly, this work enables the exploration of the optimal parameter settings for this robust design of CSEs for Li-metal batteries. This work demonstrated the exceptional performance of the ceramicized composite CSEs in suppressing Li-dendrite growth in Li|CSE|LiFePO_4_ (LFP) systems. The high compatibility of the CSEs with the Li metal anodes imparted low inner resistance and excellent capacity retention during long-duration cycling.

## 2. Experimental

### 2.1. Fabrication of CSEs

To fabricate the composite solid-state electrolytes, first, lithium bis(trifluoromethanesulfonyl)imide (LiTFSI, Alfa, Bracknell, UK, purity: 98%) and PVDF-HFP (Sigma, Saint Louis, MO, USA, molecular weight: 400,000) were homogeneously mixed using a glove box, and the N-methyl pyrrolidone (NMP, Showa, Tokyo, Japan, purity: 95%) was poured into the mixture. Highly crystalline LATP powders with an average particle size of 5 μm were supplied from Gold Carbon Co., Ltd., Taoyuan City, Taiwan. The LATP powders were synthesized by using an efficient sol-gel method, followed by thermal calcination. The LATP powders were gradually added to the solution, and the entire CSE slurry was uniformly dispersed through a planetary milling process with a rotation speed of 700 rpm for 1 h. The weight ratio of the PVDF-HFP in the CSE films was approximately 25 wt.%. The resulting LATP-containing slurry was then coated onto a polymeric membrane (polyethylene (PE) separator (Hipore AC-0881, Yan Tin Chemical Co., Ltd., Hong Kong, China) with a nominal thickness of approximately 8 μm and an uncompressed porosity of approximately 38%. The nominal weight of the PE membrane was ~4.6–4.9 g m^−2^. The thicknesses of the CSEs films casted on the PE membranes were tuned using a doctor blade (i.e., 50, 100, and 150 μm). The actual thicknesses were approximately 40, 70, and 100 μm. The above preparation process was carried out in a glove box, after which the CSEs were dehydrated at 140 °C in a vacuum oven overnight. 

### 2.2. Assembly of Li-Metal Batteries

The electrochemical characterization of the CSEs was conducted using a coin cell configuration (type: CR2032) with commercially available LFP (Ubiq Technology Co., Taoyuan, China) as the active material for the cathode electrode and Li metal as the anode material. To fabricate the LFP cathode, the LFP powders (average size of ~0.5 μm) were mixed with a binder (PVDF) and a conducting medium (Super-P, Taiwan Maxwave Co., Taipei City, Taiwan) with a 90:7:3 wt.% in an NMP solvent to form the LFP slurry. The mixture was blended using a three-dimensional mixer equipped with Zr balls for 2 h to form a uniform slurry. The resulting slurry was subsequently coated on the Al foil substrate with a doctor blade. The LFP cathode sheets were dried at 110 °C in a vacuum oven overnight. Afterward, the LFP sheets were compressed and then cut into the desired shape for the battery assembly. The surface loading of the LFP was approximately 6–7 mg cm^−2^, with an average thickness of 70 μm, and the thickness of Li metal was approximately 10 μm. The as-prepared CSEs (with PE membranes) were placed on the LFP cathode, and the entire composite sheet was tightly compressed under a pressure of approximately 200 kg cm^−2^ and then baked at 140 °C in a vacuum oven for 24 h to remove any residual organic solvent (e.g., NMP) from the CSEs. After drying, the Li|CSE|LFP batteries were assembled as the CR-2032-type coin cells in a glove box with LFP cathodes, Li-metal anodes, and CSE (i.e., LATP plus LiTFSI plus PVDF-HFP) solid-state electrolytes. 

### 2.3. Materials and Electrochemical Characterization

The morphologies of the LFP, LATP, and CSE were characterized using field-emission scanning electrode microscopy (FE-SEM, JEOL JSM-6700F, Tokyo, Japan). The crystalline structure of the LFP, LATP, CSE samples was analyzed through X-ray diffraction (XRD, Brucker D2 diffractometer with Cu target, Billerica, MA, USA). To explore the real-time performance of the as-prepared CSEs, the charge/discharge cycling experiments were performed at different C rates (which varied from 0.1 to 3 C) within the voltage range of 2.8–4.0 V at ambient temperatures. The batteries were first charged using a conventional protocol of constant current–constant voltage (i.e., different C rates to 4.0 V, with a 0.01 mA cut-off current), followed by discharging to 2.8 V at a constant current. Electrochemical impedance spectroscopy (EIS, CH Instruments 608C, Austin, TX, USA) was also conducted to quantify the polarization distribution of the coin cells assembled with the various CSEs. The EIS measurements were carried out at different potentials within the frequency range of 10 mHz to 100 kHz. 

## 3. Results and Discussion

**Figure 1:**
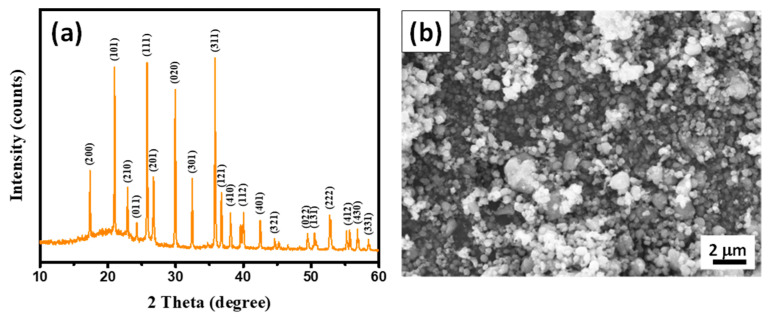
(**a**) Typical XRD pattern and (**b**) FE-SEM image of LFP powders.

Figure 1a shows typical XRD patterns of pristine LFP powders, including the characteristic peaks of crystalline LFP. The diffraction peaks within the LFP sample matched quite well with the standard orthorhombic olivine phase of LFP (JCPDS Card No.: 83-2092: *a* = 10.334 Å, *b* = 6.010 Å, and *c* = 4.693 Å) [30]. The XRD pattern confirmed that the LFP powders were highly crystalline orthorhombic olivine [31]. The FE-SEM image of the pristine LFP sample, as shown in Figure 1b, illustrates the homogeneous dispersion of the quasi-spherical powders, with an average particle size of ~500 nm.

**Figure 2:**
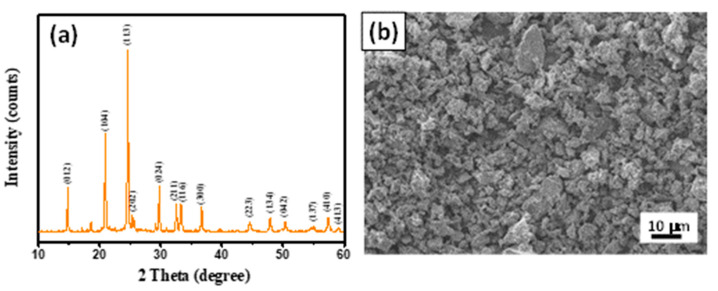
(**a**) Typical XRD pattern and (**b**) FE-SEM image of LATP powders.

In this study, the composition of the Li_1+*x*_Al*_x_*Ti_2-*x*_(PO_4_)_3_ with *x* = 0.3 (i.e., Li_1.3_Al_0.3_Ti_1.7_(PO_4_)_3_) was specifically chosen to demonstrate its potential for high-performance batteries. The LATP powders, which were made using the citric acid-assisted sol-gel synthesis method followed by calcination, possessed highly crystalline structures, as depicted in Figure 2a. The XRD pattern of the as-synthesized LATP powder can be indexed to the standard NASICON-type structure (i.e., rhombohedral lattice, Card No.: ICDD 00-035-0754). The characteristic peaks at 2*θ* = 20.8, 24.5, 29.7, 33.3, and 36.5° were assigned to the crystalline planes of (012), (104), (113), (024), and (116), respectively [32,33]. It is important to note that there is no other diffraction peak that commonly appears in the case of any impurity (e.g., AlPO_4_ [34]), revealing the high-phase purity of the synthesized LATP powders. An FE-SEM image of the LATP powders is provided in Figure 2b, showing the rectangular-shaped particles homogeneously dispersed within the structure. 

**Figure 3:**
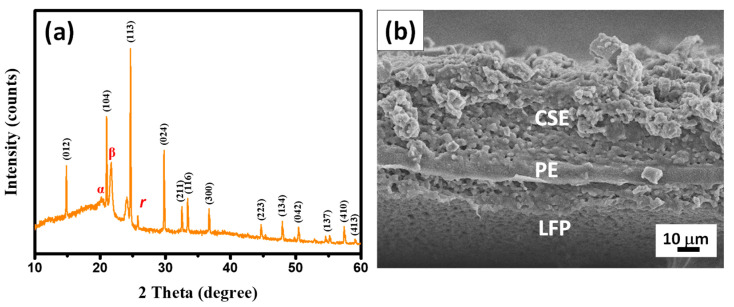
(**a**) Typical XRD pattern and (**b**) cross-sectional view of an FE-SEM image of a CSE-coated LFP cathode sheet.

The typical XRD pattern of CSEs (i.e., composition: LATP plus LiTFSI plus PVDF-HFP) is depicted in Figure 3a. According to Figure 3, the representative crystalline planes of the LATP lattices are present and the crystalline PVDF-HFP morphology can be identified at approximately 19.0, 20.6, and 26.9°, corresponding to the α, β, and γ crystalline phases of a PVDF-HFP structure, respectively [35]. To further explore the interfacial layer, a cross-sectional view FE-SEM image of the interface of the LFP cathode and the CSEs was recorded (see Figure 3b). It is clear that the CSE layer tightly covered the LFP cathode sheets, where the CSE coating formed a dense and solid film with a uniform thickness of approximately 40 μm. There was no obvious porosity, and a cavity appeared at the interface between the CSE and the LFP cathode, indicating that there was good adhesion to the electrode. To ensure the uniformity of the CSEs, the elemental mapping including Ti, P, O, Al, and F was employed to characterize the chemical distribution at the cross-sectional CSEs, as shown in the Supplementary Materials (see Figure S1). As shown in Figure S1, the colorful dots were well dispersed at the cross-sectional view of the FE-SEM images, indicating well-prepared CSEs with LATP ceramics and LiTFSI salts.

**Figure 4:**
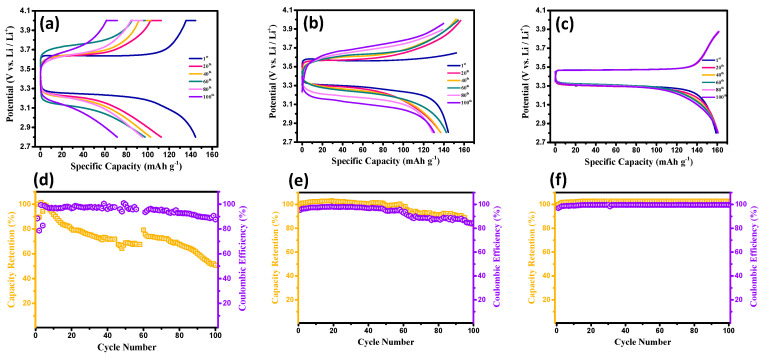
Typical charge–discharge curves and cyclic performance of the test cells equipped with different CSEs, where the LiTFSI/LATP ratios were 1.5 (**a**,**d**), 1.8 (**b**,**e**), and 2.0 (**c**,**f**).

Figure 4a–c shows typical charge–discharge curves of the coin cells equipped with the different CSEs, where the LiTFSI/LATP weight ratios were set at 1.5, 1.8, and 2.0. The Li-metal batteries were operated at 0.5 C within the potential window of 2.6–4.0 V vs. Li/Li^+^. Notably, a major flat plateau was observed, corresponding to a two-phase solid reaction of LiFePO_4_ ↔ (1 − *x*) LiFePO_4_ plus *x* FePO_4_ plus *x* Li^+^ plus *x* e^−^ [36,37], at ~3.2−3.6 V vs. Li/Li^+^, with a theoretical specific capacity of 175 mAh g^−1^. For all test cells, the charge–discharge curves were symmetric at 0.5 C, revealing the reversibility of the Li^+^ intercalation and de-intercalation [38,39,40]. In addition, as illustrated in Figure 4, the discharge capacity as a function of cycle number for the as-prepared CSEs (i.e., LiTFSI/LATP ratio: 2.0) reached as high as approximately 161 mAh g^−1^. Importantly, the electrode polarization (i.e., the potential difference between the charging and discharging plateau, Δ*E*) was vastly influenced by the LiTFSI/LATP ratio, where Δ*E* demonstrated the following order: LiTFSI/LATP ratio: 2.0 (approximately 0.25 V) < LiTFSI/LATP ratio: 1.8 (approximately 0.28 V) < LiTFSI/LATP ratio: 1.5 (approximately 0.31 V). According to this observation, a higher LiTFSI content tended to facilitate the ionic migration within the composite layer, alleviating the ionic diffusion resistance in the solid phase. 

To further explore the efficacy of the as-prepared composite solid-state electrolytes, the cyclic performance of the test cells equipped with different CSEs were analyzed at 0.5 C (see Figure 4d–f). Capacity retention and coulombic efficiency are usually employed in evaluating the cyclic stability of CSEs upon being cyclically charged/discharged. As illustrated in Figure 4, the coin cell assembled with the CSE (i.e., LiTFSI/LATP ratio: 2.0) exhibited a high-capacity retention capability (~99.6%), along with excellent coulombic efficiency (~99.3%), after 100 cycles. In contrast, the other two cell configurations displayed poor cyclic performance (e.g., low-capacity retention of approximately 50–82% after 100 cycles), confirming the critical role of the CSE configuration on the electrochemical performance of the Li-metal batteries.

**Figure 5:**
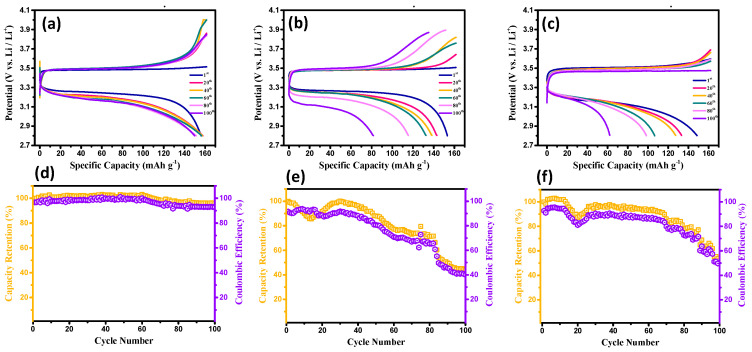
Typical charge–discharge curves and cyclic performance of the test cells equipped with CSEs (PVDF-HFP/LATP = 2.0) of 40 μm (**a**,**d**), 70 μm (**b**,**e**), and 100 μm (**c**,**f**), where the LiTFSI/LATP ratio was 1.5.

Next, the influence of CSE thickness on the electrochemical performance of the lithium-ion batteries with Li-metal anodes was explored, as shown in Figure 5. The thicknesses of the CSE films on the LFP cathodes were controlled at 40, 70, and 100 μm. The Li-metal batteries were cyclically charged and discharged for 100 cycles at 0.5 C at an ambient temperature. According to Figure 5, the reduced CSE thickness resulted in the improved cyclic performance of the coin cells. Compared to other configurations, the Li-metal batteries equipped with 40 μm-thick CSE films displayed the best cyclic stability (i.e., the capacity retention was ~96.3%) and a remarkable coulombic efficiency (~92.5%) after 100 cycles. Indeed, reducing the CSE thickness reduced the ohmic polarization. In contrast, thicker CSE layers imparted longer diffusion pathways for the Li^+^ ions. In particular, at higher C-rate operations, an increased ohmic polarization may result in the poor cyclic performance of Li-metal batteries during long-duration cycling.

**Figure 6:**
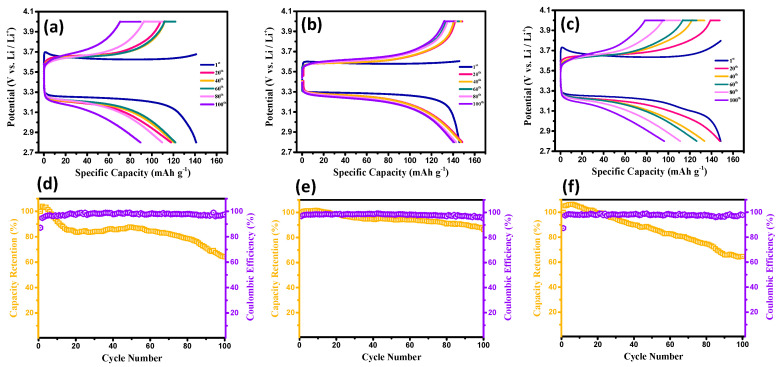
Typical charge-discharge curves and cyclic performance of the test cells equipped with CSEs (PVDF-HFP/LATP= 2.0) of 5 wt.% C (**a**,**d**), 7 wt.% C (**b**,**e**), and 15 wt.% C (**c**,**f**).

The interfacial layer between the CSEs and LFP cathodes played a critical role, affecting the rate capability and cyclic stability of the coin cells. It is generally recognized that conductive carbon particles (Super-P in this case), due to their intrinsic hydrophobicity [41,42], significantly influence the wetting property of a CSE layer. The wetting characteristic of a CSE is strongly related to the surface tension of an LFP cathode toward gel-phase composite electrolytes. To inspect the influence of surface hydrophobicity, the CSEs were coated onto three types of LFP cathodes (with three different contents of the carbon medium (5, 7, and 15 wt.%)), and the corresponding electrochemical performances were analyzed. The charge–discharge curves at 0.5 C, as well as the cyclic performances of the different Li-metal batteries, are shown in Figure 6. As clearly demonstrated in Figure 6, the LFP cathode containing 7 wt.% Super-P conductive carbon delivered the lowest polarization (approximately 0.25 V), excellent coulombic efficiency (approximately 97.5%), and higher-capacity retention (approximately 90.1%) after 100 cycles compared to other samples. This finding reflects that an unoptimized C content within the cathode sheets can easily result in the unwanted peeling of CSEs from the LFP cathode, and it can even ease the formation of the Li dendrites that lead to poor cyclic stability.

**Figure 7:**
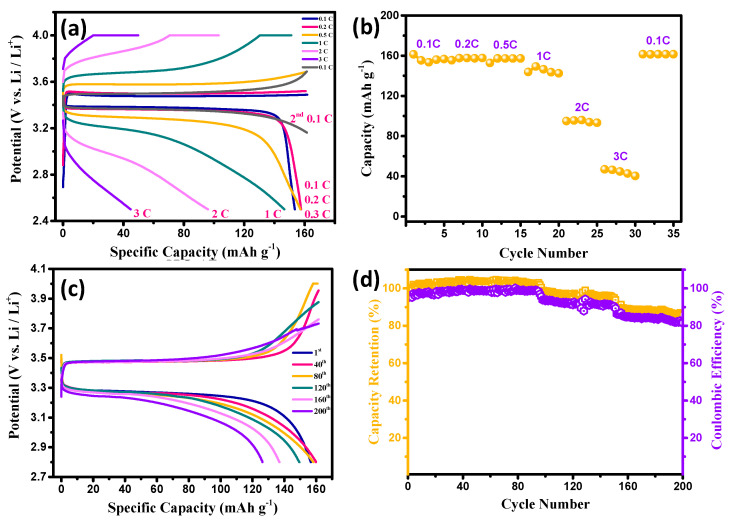
(**a**) Typical charge–discharge curves at various rates, (**b**) variations in specific capacity with C rate, (**c**) charge–discharge curves at 0.5 C, and (**d**) cyclic performance of the test cells equipped with CSEs (PVDF-HFP/LATP = 2.0, thickness = 40 μm, C content: 7 wt.%, and heat-treated temperature: 140 °C).

Considering the effects of the various design parameters on the performance of the coin cells, an optimal CSE configuration (i.e., LiTFSI/LATP ratio, thickness, and carbon content) was engineered (i.e., PVDF-HFP/LATP: 2.0, thickness: 40 μm, C content: 7 wt.%, and heat-treated temperature: 140 °C) for the fabrication of the Li-metal batteries. The typical charge–discharge curves at various rates, as well as the variations in the specific capacity with C rate, for the coin cells equipped with this optimal CSE configuration were recorded (see Figure 7). As shown in Figure 7a, the Li-metal battery was galvanostatically charged and discharged between 2.5 and 4.0 V (vs. Li/Li^+^) at different rates (i.e., 0.1, 0.2, 0.5, 1, 2, and 3 C). The performance curves shown in Figure 7 contain the typical charge/discharge plateau occurring at approximately 3.3–3.5 V at 0.1–0.5 C, indicating the presence of a two-phase Fe^3+^/Fe^2+^ redox reaction via a first-order transition between the FePO_4_ and LFP [31,39,43]. With the optimized CSE configuration, the Δ*E* value at 0.5 C was dramatically reduced to 0.12 V, significantly reducing the electrode polarization. In addition, according to Figure 7, the Li-metal batteries were robustly charged and discharged at 1–3 C. The discharge capacity as a decreasing function of the C rate is depicted in Figure 7b. Considering the discharge capacity at 0.1 C as the basis for comparison, the capacity retentions were maintained at 98.1% (0.2 C), 96.8% (0.5 C), 87.5% (1 C), 56.3% (2 C), and 25.1% (3 C). Notably, all the coin cells demonstrated 100% capacity retention at 0.1 C after completing the cycling test, indicating the superior Li^+^ reversibility through the CSE layer, even after high-rate cycling experiments. 

Figure 7c illustrates the variations in the charge-discharge curves of the coin cells at 0.5 C during extended cycling (i.e., 1–200 cycles). Based on Figure 7c, the Δ*E* value gradually increased with an increased cycle number, which was primarily due to the gradual aging of the CSE layer. However, the capacity retention remained high (i.e., 86.8%), even after 200 cycles. The variation in the capacity retention and coulombic efficiency of the Li-metal battery fabricated with the optimal CSEs is shown in Figure 7d. As clearly illustrated in Figure 7d, the Li-metal batteries were cyclically charged/discharge at 0.5 C for 200 cycles. The capacity retention was maintained at >85%, with a stable coulombic efficiency after 200 cycles. Indeed, such a robust performance was likely due to: (i) the lack of dissolution of the iron from the LFP cathode [44,45], (ii) the uniform formation of the solid electrolyte interphase layer, and (iii) the lack of a substantial formation of Li dendrites [46,47] during the long-duration cycling, confirming that this was a stable design of the CSE layer for the Li-metal batteries. However, the gradual aging of the optimal CSEs requires an in-depth investigation. This may presumably be due to a slight electrode polarization during the charge/discharge process, causing the accumulation of Li metal at the interface between the Li anode/CSE/LFP cathode. Accordingly, work regarding nanoscaled LATP, a thinner CSE layer, and a hot compression process is in progress. 

**Figure 8:**
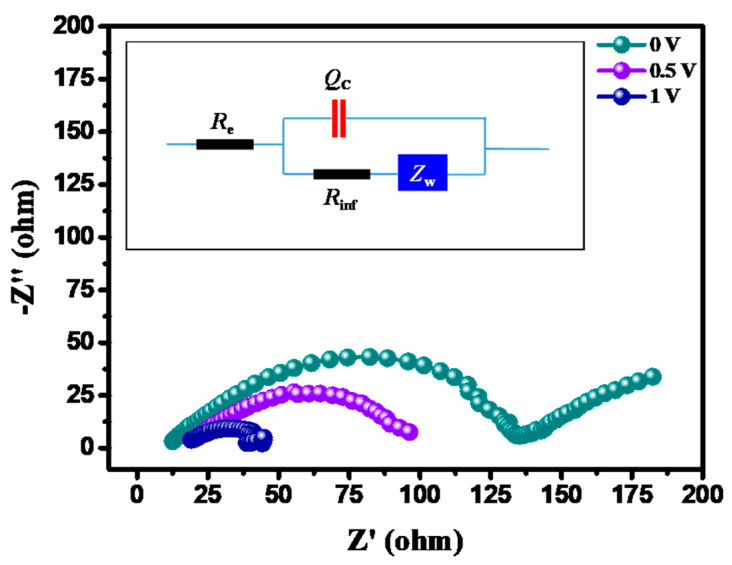
Nyquist plots of the symmetric cell fabricated with the optimal CSEs at different potentials, where the inset shows a proposed equivalent circuit. The elements, *R_e_*, *R_inf_*, *Q_C_*, and *Z_w_*, represent the resistance of the bulk electrolyte, the interfacial charge transfer resistance, the constant-phase element, and the Warburg impedance associated with the Li+ diffusion within the electrode, respectively.

To further explore the electrochemical performance of the optimized CSE layers, EIS was employed, and the impedance behaviors of the symmetric cells fabricated with the as-prepared CSEs were assessed accordingly. Figure 8 shows typical Nyquist plots of the test cells at different potentials, where all the curves intersected the x-axis at high frequency regions, followed by a depressed semicircle. According to Figure 8, the charge transfer resistance associated with the CSE film (i.e., the depressed semicircle within the Nyquist plots) demonstrated a decreasing function of the applied potential. One equivalent circuit, as shown in the inset of Figure 8, was proposed to describe the EIS behavior and to quantify the ionic conductivity (*σ*) of the CSE layer. The equivalent circuit model contained the following components: *R*_e_, *R*_inf_, *Q*_C_, and *Z*_w_, representing the resistance of the bulk electrolyte, the interfacial charge transfer resistance, the constant-phase element, and the Warburg impedance associated with the Li^+^ diffusion within the electrode, respectively [48]. The *Z*-view software package was employed to analyze the impedance spectra of the CSE layer, where the deviation between the experimentally recorded impedance spectra and the model predictions was less than 10%. The ionic conductivity was subsequently calculated using the formula *σ* = *l*/*R*_e_ *A* [49,50], where the thickness of the CSE layer (*l*) and the projected active area (*A*) were used within the formulation.

The ionic conductivity of the CSE sample remained relatively unchanged (2.83 × 10^−4^, 2.45 × 10^−4^, and 2.33 × 10^−4^ S cm^−1^ at 0, 0.5, and 1.0 V vs. Li/Li^+^, respectively), regardless of the applied potential, confirming a highly stable CSE structure. This finding confirms that the *σ* value of the as-prepared and highly optimized CSE layer was significantly enhanced via engineering the CSE configuration compared to the LATP-based electrolytes reported in the literature (i.e., those of approximately 1.11 × 10^−4^ S cm^−1^ [13]). For comparison, Tables S1 and S2 in Supplementary Materials show the calculated ionic conductivities of various CSEs in the Li-metal batteries, based on the Nyquist plots, incorporated with the proposed equivalent circuit. We observed that both a higher LiTFSI/LATP ratio and a thinner CSE thickness displayed a positive effect on the ionic conductivity, as demonstrated in the proceeding sections. This reveals that there was an optimal parameter setting in the robust design of the CSEs. Indeed, such an enhanced ionic conductivity at room temperature is largely due to the lower crystallinity of the polymeric electrolytes (i.e., PVDF-HFP) in the presence of the well-dispersed LATP particles within the hybrid heterogeneous structure, which had a critical role in boosting the ionic conductivity. Accordingly, the optimal parameters for the CSE layer demonstrated in this work can be adopted to create a three-dimensional conductive network for facilitating ionic conductivity within hybrid electrolytes while alleviating the interfacial electrolyte/electrode resistance.

## 4. Conclusions

In this work, an efficient technique was developed for fabricating high-performance CSEs containing NASICON-type LATP particles, PVDF-HFP, and LiTFSI salt for Li-metal batteries. The key design parameters (i.e., LiTFSI/LATP, CSE thickness, and carbon content) were engineered to enhance the batteries’ specific capacity, coulombic efficiency, and cyclic stability. The coin cells equipped with the highly optimized CSEs and assembled with Li-metal anodes and the LFP cathodes demonstrated high specific capacity (~160 mAh g^−1^), reduced electrode polarization (~0.12 V), and superior cyclic stability (capacity retention of 86.8%) after 200 cycles. The ionic conductivity of the optimized CSE layer reached as high as 2.83 × 10^−4^ S cm^−1^ at an ambient temperature. The remarkable cycling performance of the CSE layer demonstrated in this work was primarily due to: (i) the lack of dissolution of iron from the LFP cathode, (ii) the uniform formation of the solid electrolyte interphase layer, and (iii) the lack of substantial Li dendrite growth during the extended cycling. The framework established in this study for designing high-performance solid-state electrolytes with NASICON-type ceramic particles can be adopted to create a conductive ionic pathway for facilitating Li^+^ ionic transport within an electrolyte while alleviating the interfacial electrolyte/electrode resistance in Li-metal batteries. Accordingly, the CSE structures containing LATP powders prepared in this work can be applied for substantially boosting ionic conductivity, specific capacity, and cycle life while mitigating the interfacial resistance of the electrolyte/electrode layer, Li dendrite formation, and ionic diffusion resistance during long-duration cycling for lithium-ion batteries using Li metal as the anode electrode.

## Data Availability

The data presented in this study are available on request from the corresponding author.

## Supplementary Materials

The following supporting information can be downloaded at: https://www.mdpi.com/article/10.3390/membranes13020201/s1, Table S1: The ionic conductivities from various CSE layers in Li-metal batteries, where CSE thickness = 40 μm and C content: 7 wt.%; Table S2: The ionic conductivities from various CSE layers in Li-metal batteries, where LiTFSI/LATP ratio = 1.5 and C content: 7 wt.%; Figure S1: Cross-sectional view and elemental mapping on CSE-coated LFP cathode sheet.

## References

[1] Siller V., Morata A., Eroles M.N., Arenal R., Gonzalez-Rosillo J.C., López del Amo J.M., Tarancon A. (2021). High performance LATP thin film electrolytes for all-solid-state microbattery applications. J. Mater. Chem. A.

[2] Liao G., Mahrholz T., Geier S., Wierach P., Wiedemann M. (2018). Nanostructured all-solid-state supercapacitors based on NASICON-type Li_1.4_Al_0.4_Ti_1.6_(PO_4_)_3_ electrolyte. J. Solid State Electrochem..

[3] Soavi F., Bettini L.G., Piseri P., Milani P., Santoro C., Atanassov P., Arbizzani C. (2016). Miniaturized supercapacitors: Key materials and structures towards autonomous and sustainable devices and systems. J. Power Sources.

[4] Li Y., Wang H. (2021). Composite solid electrolytes with NASICON-type LATP and PVdF-HFP for solid-state lithium batteries. Ind. Eng. Chem. Res..

[5] Li J., Ma C., Chi M., Liang C., Dudney N.J. (2015). Solid electrolyte: The key for high-voltage lithium batteries. Adv. Energy Mater..

[6] Zheng F., Kotobuki M., Song S., Lai M.O., Lu L. (2018). Review on solid electrolytes for all-solid-state lithium-ion batteries. J. Power Sources.

[7] Mauger A., Julien C.M., Paolella A., Armand M., Zaghib K. (2019). Building better batteries in the solid state: A review. Materials.

[8] Murugan R., Thangadurai V., Weppner W. (2007). Fast lithium ion conduction in garnet-type Li_7_La_3_Zr_2_O_12_. Angew. Chem. Int. Ed..

[9] Fu K., Gong Y., Liu B., Zhu Y., Xu S., Yao Y., Luo W., Wang C., Lacey S.D., Dai J. (2017). Toward garnet electrolyte−based Li metal batteries: An ultrathin, highly effective, artificial solid-state electrolyte/metallic Li interface. Sci. Adv..

[10] Morimoto H., Awano H., Terashima J., Shindo Y., Nakanishi S., Ito N., Ishikawa K., Tobishima S.I. (2013). Preparation of lithium ion conducting solid electrolyte of NASICON-type Li_1+x_Al_x_Ti_2−x_(PO_4_)_3_ (x = 0.3) obtained by using the mechanochemical method and its application as surface modification materials of LiCoO_2_ cathode for lithium cell. J. Power Sources.

[11] DeWees R., Wang H. (2019). Synthesis and properties of NASICON type LATP and LAGP solid electrolytes. ChemSusChem.

[12] Paolella A., Zhu W., Xu G.-L., La Monaca A., Savoie S., Girard G., Vijh A., Demers H., Perea A., Delaporte N. (2020). Understanding the reactivity of a thin Li_1.5_Al_0.5_Ge_1.5_(PO_4_)_3_ solid-state electrolyte toward metallic lithium anode. Adv. Energy Mater..

[13] Chen S.-Y., Hsieh C.-T., Zhang R.-S., Mohanty D., Ashraf Gandomi Y., Hung I.-M. (2022). Hybrid solid state electrolytes blending NASICON-type Li_1+x_Al_x_Ti_2–x_(PO_4_)_3_ with poly(vinylidene fluoride-co-hexafluoropropene) for lithium metal batteries. Electrochim. Acta.

[14] Liu Z., Fu W., Payzant E., Yu X., Wu Z., Dudney N., Kiggans J., Hong K., Rondinone A., Liang C. (2013). Anomalous high ionic conductivity of nanoporous beta-Li_3_PS_4_. J. Am. Chem. Soc..

[15] Yu C., Ganapathy S., de Klerk N.J., Roslon I., van Eck E.R., Kentgens A.P., Wagemaker M. (2016). Unravelling Li-ion transport from picoseconds to seconds: Bulk versus interfaces in an argyrodite Li_6_PS_5_Cl-Li_2_S all-solid-state Li-ion battery. J. Am. Chem. Soc..

[16] Ziolkowska D.A., Arnold W., Druffel T., Sunkara M., Wang H. (2019). Rapid and economic synthesis of a Li_7_PS_6_ solid electrolyte from a liquid approach. ACS Appl. Mater. Interfaces.

[17] Arnold W., Buchberger D.A., Li Y., Sunkara M., Druffel T., Wang H. (2020). Halide doping effect on solvent-synthesized lithium argyrodites Li_6_PS_5_X (X= Cl, Br, I) superionic conductors. J. Power Sources.

[18] Han L., Hsieh C.-T., Mallick B., Li J., Ashraf Gandomi Y. (2021). Recent progress and future perspective on atomic layer deposition to prepare/modify solid-state electrolytes and interface between electrodes for next-generation lithium batteries. Nanoscale Adv..

[19] Key B., Schroeder D.J., Ingram B.J., Vaughey J.T. (2012). Solution-based synthesis and characterization of lithium-ion conducting phosphate ceramics for lithium metal batteries. Chem. Mater..

[20] Liu Y., Li B., Kitaura H., Zhang X., Han M., He P., Zhou H. (2015). Fabrication and performance of all-solid-state Li-air battery with SWCNTs/LAGP cathode. ACS Appl. Mater. Interfaces.

[21] Zhang P., Matsui M., Takeda Y., Yamamoto O., Imanishi N. (2014). Water-stable lithium ion conducting solid electrolyte of iron and aluminum doped NASICON-type LiTi_2_(PO_4_)_3_. Solid State Ion..

[22] Mertens A., Yu S., Schon N., Gunduz D.C., Tempel H., Schierholz R., Hausen F., Kungl H., Granwehr J., Eichel R. (2017). Superionic bulk conductivity in Li_1.3_Al_0.3_Ti_1.7_(PO_4_)_3_ solid electrolyte. Solid State Ion..

[23] Commarieu B., Paolella A., Daigle J.-C., Zaghib K. (2018). Toward high lithium conduction in solid polymer and polymer-ceramic batteries. Curr. Opin. Electrochem..

[24] Li Y., Arnold W., Thapa A., Jasinski J.B., Sumanasekera G., Sunkara M., Druffel T., Wang H. (2020). Stable and flexible sulfide composite electrolyte for high-performance solid-state lithium batteries. ACS Appl. Mater. Interfaces.

[25] Lin D., Liu W., Liu Y., Lee H.R., Hsu P.-C., Liu K., Cui Y. (2016). High ionic conductivity of composite solid polymer electrolyte via in situ synthesis of monodispersed SiO_2_ nanospheres in poly(ethylene oxide). Nano Lett..

[26] Choudhury S., Stalin S., Vu D., Warren A., Deng Y., Biswal P., Archer L.A. (2019). Solid-state polymer electrolytes for high-performance lithium metal batteries. Nat. Commun..

[27] Han L., Lehmann M., Zhu J., Liu T., Zhou Z., Tang X., Hsieh C.-T., Sokolov A.P., Cao P., Chen X. (2020). Recent developments and challenges in hybrid solid electrolytes for lithium-ion batteries. Front. Energy Res..

[28] Subadevi R., Sivakumar M., Rajendran S., Wu H.C., Wu N.L. (2011). Development and characterizations of PVdF-PEMA gel polymer electrolytes. Ionics.

[29] Karuppasamy K., Reddy P.A., Srinivas G., Tewari A., Sharma R., Shajan X.S., Gupta D. (2016). Electrochemical and cycling performances of novel nonafluorobutanesulfonate (nonaflate) ionic liquid based ternary gel polymer electrolyte membranes for rechargeable lithium ion batteries. J. Membr. Sci..

[30] Wang M., Xue Y., Zhang K., Zhang Y. (2011). Synthesis of FePO_4_·2H_2_O nanoplates and their usage for fabricating superior high-rate performance LiFePO_4_. Electrochim. Acta.

[31] Hsieh C.-T., Pai C.-T., Chen Y.-F., Chen I.-L., Chen W.-Y. (2014). Preparation of lithium iron phosphate cathode materials with different carbon contents using glucose additive for Li-ion batteries. J. Taiwan Inst. Chem. Eng..

[32] Liang Y., Lin Z., Qiu Y., Zhang X. (2011). Fabrication and characterization of LATP/PAN composite fiber-based lithium-ion battery separators. Electrochim. Acta.

[33] Kumar J., Kichambare P., Rai A.K., Bhattacharya R., Rodrigues S., Subramanyam G.A. (2016). A high performance ceramic-polymer separator for lithium batteries. J. Power Sources.

[34] Watzig K., Rost A., Langklotz U., Matthey B., Schilm J. (2016). An explanation of the microcrack formation in Li_1.3_Al_0.3_Ti_1.7_(PO_4_)_3_ ceramics. J. Eur. Ceram. Soc..

[35] Arbi K., Mandal S., Rojo J.M., Sanz J. (2002). Dependence of ionic conductivity on composition of fast ionic conductors Li_1+x_Ti_2-x_Al_x_(PO_4_)_3_, 0 ≤ x ≤ 0.7. A parallel NMR and electric impedance study. Chem. Mater..

[36] Xu G., Li F., Tao Z., Wei X., Liu Y., Li X., Ren Z., Shen G., Han G. (2014). Monodispersed LiFePO_4_@C core–shell nanostructures for a high power Li-ion battery cathode. J. Power Sources.

[37] Hsieh C.-T., Liu J.-R., Juang R.-S., Lee C.-E., Chen Y.-F. (2015). Microwave synthesis of copper network onto lithium iron phosphate cathode materials for improved electrochemical performance. Mater. Chem. Phys..

[38] Hsieh C.-T., Chen I.-L., Chen W.-Y., Wang J.-P. (2012). Synthesis of iron phosphate powders by chemical precipitation route for high-power lithium iron phosphate cathodes. Electrochim. Acta.

[39] Hsieh C.-T., Pai C.-T., Chen Y.-F., Yu P.-Y., Juang R.-S. (2014). Electrochemical performance of lithium iron phosphate cathodes at various temperatures. Electrochim. Acta.

[40] Ahsan Z., Ding B., Cai Z., Wen C., Yang W., Ma Y., Zhang S., Song G., Javed M.S. (2021). Recent progress in capacity enhancement of LiFePO_4_ cathode for Li-ion batteries. J. Electrochem. Energy Convers. Storage.

[41] Hsieh C.-T., Chen W.-Y., Wu F.-L. (2008). Fabrication and superhydrophobicity of fluorinated carbon fabrics with micro/nanoscaled two-tier roughness. Carbon.

[42] Hsieh C.-T., Teng H., Chen W.-Y., Cheng Y.-S. (2010). Synthesis, characterization, and electrochemical capacitance of amino-functionalized carbon nanotube/carbon paper electrodes. Carbon.

[43] Nien Y.H., Carey J.R., Chen J.S. (2009). Physical and electrochemical properties of LiFePO_4_/C composite cathode prepared from various polymer-containing precursors. J. Power Sources.

[44] Wu H., Wu H., Lee E., Wu N. (2010). High-temperature carbon-coated aluminum current collector for enhanced power performance of LiFePO_4_ electrode of Li-ion batteries. Electrochem. Commun..

[45] Chang H.H., Chang C.C., Su C.Y., Wu H.C., Yang M.H., Wu N.L. (2008). Effects of TiO_2_ coating on high-temperature cycle performance of LiFePO_4_-based lithium-ion batteries. J. Power Sources.

[46] Jaumaux P., Liu Q., Zhou D., Xu X., Wang T., Wang Y., Kang F., Li B., Wang G. (2020). Deep-eutectic-solvent-based self-healing polymer electrolyte for safe and long-life lithium-metal batteries. Angew. Chem..

[47] Sun J., Yao X., Li Y., Zhang Q., Hou C., Shi Q., Wang H. (2020). Facilitating interfacial stability via bilayer heterostructure solid electrolyte toward high-energy, safe and adaptable lithium batteries. Adv. Energy Mater..

[48] Mishra M., Hsu C.W., Rath P.C., Patra J., Lai H.Z., Chang T.L., Wang C.-Y., Wu T.Y., Lee T.-C., Chang J.K. (2020). Ga-doped lithium lanthanum zirconium oxide electrolyte for solid-state Li batteries. Electrochim. Acta.

[49] Chao C.H., Hsieh C.T., Ke W.J., Lee L.W., Lin Y.F., Liu H.W., Gu S., Fu C.C., Juang R.S., Mallick B.C. (2021). Roll-to-roll atomic layer deposition of titania coating on polymeric separators for lithium ion batteries. J. Power Sources.

[50] Prasanna K., Subburaj T., Lee W.J., Lee C.W. (2014). Polyethylene separator: Stretched and coated with porous nickel oxide nanoparticles for enhancement of its efficiency in Li-ion batteries. Electrochim. Acta.

