# Supplementary material for: Sodium Super Ionic Conductor-Type Hybrid Electrolytes for High Performance Lithium Metal Batteries
Source: Membranes (Basel). 2023 Feb 6;13(2):201. doi: 10.3390/membranes13020201 (PMC9960259; doi:10.3390/membranes13020201)
Supplement: Supplementary file 1 [file membranes-13-00201-s001.zip › membranes-2169364-supplementary.pdf]

membranes

Supplementary Materials

Sodium Super Ionic Conductor-Type Hybrid Electrolytes for High Performance Lithium Metal Batteries

Po-Yu Sung [1], Mi Lu [2], Chien-Te Hsieh [1,3,*], Yasser Ashraf Gandomi [4,*], Siyong Gu [2] and Wei-Ren Liu [5,*]

[1] Department of Chemical Engineering and Materials Science, Yuan Ze University, Taoyuan 32003, Taiwan
[2] Key Laboratory of Functional Materials and Applications of Fujian Province, Xiamen University of Technology, Xiamen 361024, China
[3] Department of Mechanical, Aerospace, and Biomedical Engineering, University of Tennessee, Knoxville, TN 37996, USA
[4] Department of Chemical Engineering, Massachusetts Institute of Technology, Cambridge, MA 02142, USA
[5] Department of Chemical Engineering, Chung Yuan Christian University, Taoyuan 32023, Taiwan
* Correspondence: cthsieh@saturn.yzu.edu.tw (C.-T.H.); ygandomi@mit.edu (Y.A.G.); wrliu1203@gmail.com (W.-R.L.)

Table S1. The ionic conductivities from various CSE layers in Li-metal batteries, where CSE thickness = 40 μm and C content: 7 wt.%.

LiTFSI/LATP ratio	Ionic conductivity (S cm^{-1})
1.5	1.15×10^{-4}
1.8	1.83×10^{-4}
2.0	2.83×10^{-4}

Table S2. The ionic conductivities from various CSE layers in Li-metal batteries, where LiTFSI/LATP ratio = 1.5 and C content: 7 wt.%.

CSE thickness (μm)	Ionic conductivity (S cm^{-1})
40	1.15×10^{-4}
70	9.98×10^{-5}
100	5.01×10^{-5}

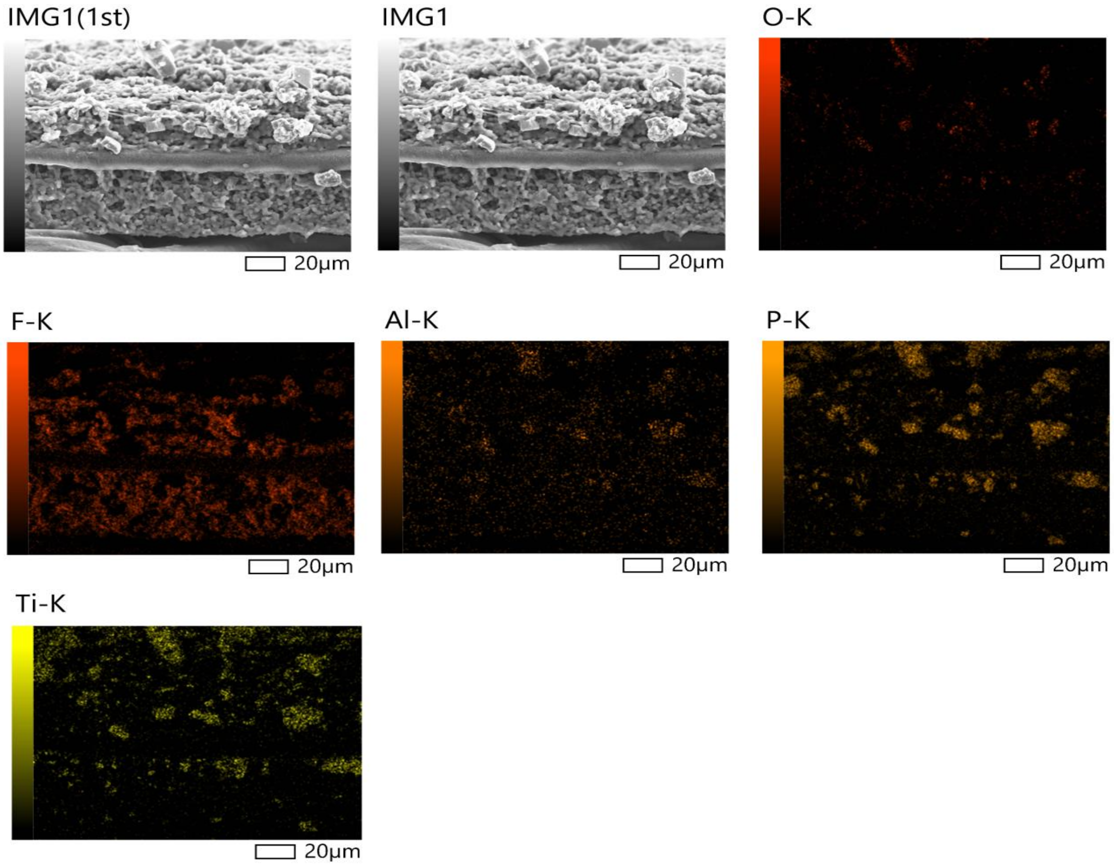

Figure S1. Cross-sectional view and elemental mapping on CSE-coated LFP cathode sheet.
